# Structural and Molecular Properties of Insect Type II Motor Axon Terminals

**DOI:** 10.3389/fnsys.2018.00005

**Published:** 2018-03-19

**Authors:** Bettina Stocker, Christina Bochow, Christine Damrau, Thomas Mathejczyk, Heike Wolfenberg, Julien Colomb, Claudia Weber, Niraja Ramesh, Carsten Duch, Natalia M. Biserova, Stephan Sigrist, Hans-Joachim Pflüger

**Affiliations:** ^1^Institute of Biology, Neurobiology, Freie Universität Berlin, Berlin, Germany; ^2^Institute of Biology, Genetics, Freie Universität Berlin, Berlin, Germany

**Keywords:** insects, neuromodulation, axonal terminals, biogenic amines, cytomatrix proteins

## Abstract

A comparison between the axon terminals of octopaminergic efferent dorsal or ventral unpaired median neurons in either desert locusts (*Schistocerca gregaria*) or fruit flies (*Drosophila melanogaster*) across skeletal muscles reveals many similarities. In both species the octopaminergic axon forms beaded fibers where the boutons or varicosities form type II terminals in contrast to the neuromuscular junction (NMJ) or type I terminals. These type II terminals are immunopositive for both tyramine and octopamine and, in contrast to the type I terminals, which possess clear synaptic vesicles, only contain dense core vesicles. These dense core vesicles contain octopamine as shown by immunogold methods. With respect to the cytomatrix and active zone peptides the type II terminals exhibit active zone-like accumulations of the scaffold protein Bruchpilot (BRP) only sparsely in contrast to the many accumulations of BRP identifying active zones of NMJ type I terminals. In the fruit fly larva marked dynamic changes of octopaminergic fibers have been reported after short starvation which not only affects the formation of new branches (“*synaptopods*”) but also affects the type I terminals or NMJs via octopamine-signaling (Koon et al., 2011). Our starvation experiments of *Drosophila*-larvae revealed a time-dependency of the formation of additional branches. Whereas after 2 h of starvation we find a decrease in “*synaptopods*”, the increase is significant after 6 h of starvation. In addition, we provide evidence that the release of octopamine from dendritic and/or axonal type II terminals uses a similar synaptic machinery to glutamate release from type I terminals of excitatory motor neurons. Indeed, blocking this canonical synaptic release machinery via RNAi induced downregulation of BRP in neurons with type II terminals leads to flight performance deficits similar to those observed for octopamine mutants or flies lacking this class of neurons (Brembs et al., 2007).

## Introduction

Vertebrate skeletal muscle is innervated by excitatory cholinergic motor neurons. By contrast, insect skeletal muscle is innervated by glutamatergic excitatory motor neurons, and may receive additional innervation by inhibitory neurons and neuromodulatory neurons (Usherwood, 1975; Wolf and Lang, 1994; Pflüger and Duch, 2011). Among the neuromodulatory neurons which supply insect muscles those releasing the biogenic amine octopamine are the most prominent. However, peptides such as allatostatin (Kreissl et al., 1999) or insulin-like peptide (Gorczyca et al., 1993) were also described to be released onto a few specialized muscles. As insect muscle fibers do not seem to produce action potentials, the motor axons have to build neuromuscular junctions (NMJs) in regular distances along the muscle fiber in order to ensure sufficient depolarization along the entirety of the fiber, and thus proper contractions (Usherwood, 1967, 1975; Fourtner, 1981; Peron et al., 2009). In *Drosophila* larvae, the axon terminals of excitatory glutamatergic motor neurons form NMJs and were named type I terminals (Keshishian et al., 1996; Feeney et al., 1998; Prokop, 2006; Prokop and Meinertzhagen, 2006), and further subdivided into type Ib and Is terminals (Atwood et al., 1993; Jia et al., 1993; Choi et al., 2004; He et al., 2009). In addition, some special peptidergic type III terminals were described (Anderson et al., 1988; Cantera and Nässel, 1992; Gorczyca et al., 1993; Zhong and Peña, 1995; Budnik, 1996), but the majority of axonal terminals of neuromodulatory neurons including octopaminergic neurons (Atwood et al., 1993; Monastirioti et al., 1995) consists of type II terminals. In other insects these aminergic axons were described as “beaded fibers” because of the regular occurrence of varicosities or boutons along their length. With respect to the vesicles found within these different terminals, those of the type I were described as round and clear with a diameter of 40–50 nm (Atwood et al., 1993; Jia et al., 1993), with differentiation into Ib and Is. The type II terminals were described in containing clear elliptical and dense core vesicles of 100–150 nm in diameter (Atwood et al., 1993). Table 1 provides data on some properties of the different terminals on larval *Drosophila* muscle.

**Table 1:** Properties of types of terminals on larval *Drosophila* muscle based upon the following references: Anderson et al. (1988), Johansen et al. (1989), Budnik and Gorczyca (1992), Cantera and Nässel (1992), Jia and Budnik (1992), Atwood et al. (1993), Gorczyca et al. (1993), Jia et al. (1993), Zhong and Peña (1995) and Prokop et al. (1996).

Terminal type	Bouton size diameter	Vesicle type	Vesicle size	Transmitter
Type I	3–5 μm	Clear vesicles (CV)	40–50 nm; 44 ± 0.3 nm	Glutamate
Type I	3.1 ± 1.6 μm		30–50 nm	
	max. 8 μm			
Type Ib	2–5 μm	CV		
Type Is	1–3 μm	CV		
Type II	1–2 μm	Dense core vesicles (DCV)	100–150 nm	Octopamine
Type II	1.4 ± 0.6 μm			
		Dark DCV	73 ± 2 nm	
		Intermediate and light DCV	108 ± 6 nm; 97 ± 1 nm	
		Small (translucent) CV	33.0 ± 0.5 nm;	
Type III	1–3 μm			Proctolin
				Insulin-like-peptide
				PACAP
				Leucokinin I

Octopaminergic neurons which form beaded fiber axons and type II like terminals were first recognized by Plotnikova (1969) in locusts and later extensively studied by Hoyle (1974, 1978 for a review, see Bräunig and Pflüger, 2001). These axons arise from a specific class of unpaired median neurons with bilaterally symmetrical axons and either dorsal (DUM-neurons) or ventral (VUM-neurons) cell bodies (Watson, 1984). The population of DUM/VUM-neurons can be further divided into subpopulations which are differentially recruited in different motor behaviors such as jump, walking or flying (Burrows and Pflüger, 1995; Baudoux et al., 1998; Duch and Pflüger, 1999; Duch et al., 1999; Mentel et al., 2008; Pflüger and Duch, 2011) or crawling (Johnston et al., 1999). The release of octopamine from type II terminals onto insect skeletal muscle has multiple effects: first, it produces a small (up to 10%) increase in twitch tension and, more significantly, an increase in relaxation rate of skeletal muscle (Evans and O’Shea, 1977; O’Shea and Evans, 1979; Ormerod et al., 2013). In contrast, myogenic contractions of specialized skeletal muscle-bundles are inhibited (Evans and O’Shea, 1978). Second, these octopaminergic neurons also have metabolic functions because they are involved in regulating (boosting) glycolysis (Mentel et al., 2003). If locust muscles have to rely on lipid metabolism, for example during flight, these neurons are switched off (Duch and Pflüger, 1999; Mentel et al., 2003). By contrast, shortly before flight octopamine release may prepare the flight power muscles for high glycolytic rates during take-off (Pflüger and Duch, 2011). Accordingly, the octopaminergic system is suggested to prime the whole organism for “soon-to-come dynamic action” and skeletal muscles are, of course, a key target (Orchard et al., 1993; Bräunig and Pflüger, 2001; Pflüger and Duch, 2011).

Octopamine is also known to be an important modulator in the central nervous system, for example in activating the CPG for flight (Wilson, 1961; Stevenson and Kutsch, 1987, 1988), and recruiting motor neurons of other than flight muscles (FMs) to the flight rhythm (Rillich et al., 2013). Correspondingly, octopamine deficient adult fruit flies have severe deficiencies in their flight performance with significantly shorter flight durations than control flies (Brembs et al., 2007). It is, however, still unclear whether this is a peripheral or central effect. Octopamine-release in the thoracic ganglia is either mediated by descending neurons, for example descending DUM/VUM-neurons of the SOG exclusively (Bräunig and Burrows, 2004; Cholewa and Pflüger, 2009), or by the dendritic processes of DUM/VUM-neurons which may also release octopamine although ultrastructural studies only revealed clear input synapses (Pflüger and Watson, 1995). On the other hand, the dendritic processes of all DUM/VUM-neurons are labeled by tyramine- and octopamine-antibodies (Kononenko et al., 2009) and also bruchpilot (BRP^NC82^, this study Supplementary Figure S1). In addition, octopamine has also been reported to affect target cell metabolism (Mentel et al., 2003; Pflüger and Duch, 2011). At present it remains unresolved what the relative contributions of the various reported effects of octopamine on central and peripheral excitability, structure, and metabolism are.

There is also a link between hunger and stress and the octopaminergic system (Wicher, 2007; Kononenko et al., 2009). In *Drosophila* larvae, the axons of octopaminergic VUM-neurons form additional *synaptopods*, defined as filipodia-like extensions (Koon et al., 2011) or small axonal sprouts (see also Supplementary Figure S4), after a very short period of starvation and, in addition, the NMJ of excitatory type I terminals itself is also affected by this (Koon et al., 2011). Different octopamine receptors are involved in the fine control of these regulatory processes of type I and type II terminals (Koon and Budnik, 2012). Therefore, it is now clear that the octopaminergic neurons play an important role for adaptive responses in multiple behavioral contexts. The aim of our study was to provide, from a comparative view, a description of structural, molecular, and functional specializations of type II terminals in locusts and fruit flies.

## Materials and Methods

### Locust Care

Individuals of both sexes of adult desert locusts, *Schistocerca gregaria* (Forskal) were taken from our crowded colony maintained in Berlin. Animals were kept in a constant 12 h light/dark cycle at a temperature of 28°C. If not stated otherwise, the locusts were anesthetized by chilling at 4°C for at least 30 min prior to experiments.

### Fruit Fly Care for Anatomical Studies

Fruit flies (*Drosophila melanogaster*) were raised at 24°C and 60% relative humidity with a 14 h:10 h light-dark cycle on cornmeal based food, following the Würzburg recipe (Guo et al., 1996). Genetic crosses were performed according to standard procedures (Sigrist et al., 2003). All experiments were performed with heterozygous 3–5 day old male F1 progeny of homozygous parental lines. Genetic lines used in this study were not outcrossed to a reference strain with a specific genetic background. For some of the experiments transgenic fruit flies TDC2gal4 × UASCd8GFP were used to label all neurons which synthesize tyramine and octopamine from tyrosine (Monastirioti et al., 1995).

### Dissections of Animals

#### Antero-/Retrograde Nerve Stainings in Locusts

Adult locusts (*n* = 6) were mounted laterally in plasticine to expose the thoracic pleura. The motor nerve N4D4 innervating the pleuroaxillary flight steering muscle (M85/M114) of the meso- or metathorax was exposed by: (i) cutting a small window into the cuticle of the posterior pleuron of the second or third thoracic segment; and then (ii) removing the dorsoventral depressor muscle (M129). The intact nerve was carefully cut and either the distal (for anterograde staining) or the proximal end (for retrograde staining) was placed in a vaseline pool which subsequently was filled with dH_2_O. After 10 min the water was replaced by neurobiotin (Neurobiotin tracer, Vector Laboratories) solution (10% in dH_2_O). The pool and part of the exposed dissection were sealed with vaseline, and animals were placed in a moist chamber for 10–24 h at 4°C to enable dye diffusion. Prior to fixation, muscles or central ganglia were excised together with their attachment sites at the pleural ridge and the axillary sclerite, and transferred to a Sylgard dish (Silicone elastomere kit, Dow Corning) filled with isotonic locust saline (150 mM NaCl, 5 mM KCl, 5 mM CaCl_2_, 2 mM MgCl_2_, 10 mM HEPES, pH 7.4). In case of retrograde staining, the thoracic ganglia were isolated and cleansed of connective tissue, fat and trachea, and also pinned to a Sylgard dish. For subsequent tissue fixation and further processing see following paragraphs. The nomenclature of nerves and muscles is used according to Snodgrass (1929) and Campbell (1961).

#### Dissections of Fruit Fly (*Drosophila*) Larvae and Adults

*Drosophila* 3rd larval instars were kept on ice, mounted in a Sylgard dish dorsal side up and cut open by a dorsal medial incision. The preparation was carefully outlined by pins (“filet”-preparation), covered by cold *Drosophila*-saline (HL3, pH 7.2; all mM, 70 NaCl, 5 KCl, 20 MgCl_2_, 10 NaHCO_3_, 5 Trehalose, 115 Sucrose, 5 HEPES), and the gut removed.

Adult flies were also kept on ice, and before mounting all legs and wings were removed. The flies were mounted dorsal side up, covered by *Drosophila*-saline and opened by a dorsal medial incision and carefully pinned to a Sylgard dish. Then guts and reproductive organs together with any loose fat were removed to expose the ventral nerve cord and thoracic muscular system.

#### Immunocytochemistry of Locust Muscle

Dissected adult locust muscles were fixed in 1% glutaraldehyde (GA) and 2% paraformaldehyde (PFA) in 0.1 M PBS (phosphate buffered saline, pH 7.4) for about 3 h. After rinsing in PBS for 2 h and dehydration by means of an ascending ethanol series (50%, 70%, 90%, 100%; 10 min each) the preparations were clarified in xylene (Merck) and subsequently rehydrated (100%, 90%, 70%, 50%; 10 min each). In order to reduce unspecific staining, 1% sodium borohydride (Merck) in PBS was applied for 10 min followed by incubation in 1% Triton X-100 (TX, Sigma) in PBS (2 h). For 1–2 h the preparations were preincubated in 10% normal goat serum (NGS, ISN Biomedicals) in 1% TX in PBS to block unspecific binding sites. Primary antibodies used were: (i) anti-synapsin I (SynORF, 3C11, kindly provided by Prof. Erich Buchner, Würzburg); and (ii) anti-NC82 (kindly provided by Prof. Stephan Sigrist, Berlin) both from mouse, applied at a dilution of 1:7 (SynI) and 1:100 (NC82); (iii) a monoclonal octopamine antibody (anti-octopamine from mouse, Bioscience, Jena, see Dacks et al., 2005; and Kononenko et al., 2009), 1:1000; and (iv) a polyclonal antibody to tyramine (anti-tyramine from rabbit, Chemicon; see Kononenko et al., 2009), 1:500. To shield samples from fungal infection, 0.02% sodium acid was added to the 1% TX 1% NGS antiserum solution. Binding of antibodies occurred at 4°C on a shaker for at least 3 days, followed by incubation in 1% TX in PBS for approximately 2 h. Secondary antibodies, Cy2- or Cy3-conjugated goat anti-mouse (Dianova), for synapsin I, NC82 and octopamine, Cy3-conjugated streptavidin (Dianova) for amplification of neurobiotin, and Cy5-conjugated goat anti-rabbit (Dianova) for tyramine (all diluted at 1:200) were added and the preparations were kept overnight at room temperature (RT). After repeated washing in PBS for 2 h the preparations were dehydrated (50%, 70%, 90%, 100%; 10 min each) and finally mounted in methyl-salicylate to a microscope slide and sealed with a cover slip (Merck).

#### Immunocytochemistry of Fruit Fly (*Drosophila*) Muscle

Larval body wall muscles and adult flight muscles (DLMs) in 10 animals each were stained with the following antibodies: anti-GFP, anti-brp (BRP), and anti-HRP (Horseradish peroxidase). In larvae, *n* = 3, body wall muscles were stained with anti-tyramine and anti-octopamine to reveal labeling in type II terminals.

For anti-GFP, anti-brp and anti-HRP the following steps were applied: the dissected larvae were covered for 60 min in 4% PFA in 0.1 M phosphate buffered saline (PBS, pH 7.4) to fix the preparations. The animals were washed overnight in 0.1 M PBS (pH 7.4) while placing the Sylgard dish inside a dark cold room at 4°C. The next day, they were washed in 0.1 M PBS + 0.5% Triton-X (TX) for 160 min while changing the solution every 20 min. For pre-incubation the dissected animals were covered in 10% NGS in 0.1 M PBS + 0.5 TX for 60 min. Then, they were washed in 1960 μl 0.1 M PBS + 0.3% TX + 20 μl NGS + 20 μl of the primary antibody anti-green-fluorescent-protein rabbit (anti-GFP; 1:100) for 2 days in a dark cold room at 4°C. Subsequently, preparations were washed in 0.1 M PBS + 0.3% TX for 120 min and the solution was changed every 15 min. For the secondary antibody staining, the animals were washed in 40 μl goat anti rabbit Alexa 488 Cy2 (1:100) + 0.2 μl Atto 565 Phalloidin + 4 μl anti-HRP Cy5 (1:500) in 1956 μl 0.1 M PBS + 0.3% TX overnight and protected from light. The next day, specimen were washed for 90 min in 0.1 M PBS with the solution being exchanged every 15 min. After that, the insect pins were carefully removed from the animals and each preparation was placed in Dianova IS mounting medium on a thin microscope slide which was then sealed with a 20 × 20 mm cover slip and stored at 4°C protected from light until confocal imaging.

For anti-tyramine and anti-octopamine staining of larval body wall muscles the above mentioned recipe for locust muscle was used (*n* = 3, see also Kononenko et al., 2009).

### Confocal Microscopy of Locust Muscle

#### Image Acquisition

Immunofluorescent labels were visualized and scanned with a confocal scanning microscope (TCS SP2, Leica, Germany). Scanning of muscle stainings was performed by using either a HC PL 10×/0.5, a HC PL 20×/0.7 imm, a HC PL 40×/1.4 imm or a HC PL 63×/1.52 imm objective at a maximal zoom factor of 3. Signals from fluorophores were detected in serial stacks of appropriate slice numbers, and at an image resolution of 1024 × 1024 pixels. For sample stack generation of stained muscle tissue three different regions per muscle (proximal, medial, distal) were chosen and stacks of constant dimension and magnification were scanned (x, y 0.15 μm, step size 1 μm), at, as far as possible, constant laser intensity. Excitation of fluorescent dyes was enabled by using three different laser lines: an Ar/Kr-Laser at 488 nm and two He/Ne-Lasers at either 543 nm or 633 nm. Images were scanned in a sequential mode to confine cross-excitation of different dyes. For analysis image stacks were processed with the software package Amira 4.1.1 (TGS, Mercury Computer Systems, Mérignac, France).

### Ultrastructure and Anti-octopamine Immunogold-Staining of Locust and Fruit Fly Muscle

#### Electron Microscopy

For the normal ultrastructure of muscles, adult desert locust* Schistocerca gregaria* (*n* = 11) tissue was prefixed in 2.5% GA in 0.1 M Na-cacodylate buffer (pH 7.4), for 3–7 min after dissection of the animal, and then fixed for 1–2 h at RT in the same fixer solution. After fixation, specimen were washed 3 × 15 min in 0.1 M PBS (pH 7.4) at RT, followed by fixation in 1% OsO_4_ in 0.1 M PBS for 1 h at RT. Then specimens were dehydrated in an ascending ethanol series (30%, 50%, 70%, 96%, 100%, 2 × 10 min each), followed by 2 × 15 min propylene oxide and 2 × 15 min acetonitrile. Specimen were then left in a mixture of acetonitrile and epoxy resin (EMbed 812), 2:1, overnight (up to 17 h) and then finally embedded in fresh resin (9 h at RT, over-night at 37°C, 48 h at 60°C) for polymerization.

To investigate the ultrastructure of wild type fruit fly (*Drosophila)* larva body wall (*n* = 5) and adult FMs (*n* = 7), specimen were fixed in 2% GA in 0.1 M PBS + 0.1 M sucrose (pH 7.4), 2 h; washed in the same buffer and post-fixed in 1% OsO_4_ in 0.1 M PBS, pH 7.4, 80 min; washed in dH_2_O (3 × 5 min) and contrasted in 4% uranyl acetate in H_2_O, 1 h, on a rotor, and rinsed again in dH_2_O (3 × 5 min). Then, specimen were dehydrated in an ascending ethanol series and acetone, and finally embedded in epoxy resin (EMbed 812, ElectronMicroscopy Sciences, Hatfield, PA, USA). Series of ultrathin sections (50–70 nm) were cut on a Leica EM KMR2 ultra-microtome with a diamond knife, mounted on formvar-coated slots, stained with 4% uranyl acetate and 0.4% lead citrate (in dH_2_O), and examined/viewed with a Philips 208 transmission electron microscope, operated at an accelerating voltage of 80 kV.

#### Immuno-Gold Staining of Locust and Fruit Fly (*Drosophila*) Muscle

For immunogold-studies, muscles of adult locusts (*Schistocerca*, *n* = 9) and larval (*n* = 4) and adult (*n* = 5) *fruit flies (Drosophila)* were examined. For locusts, the same fixation was used as mentioned in the previous chapter. For *Drosophila* we used fixation in 1% GA + 2% PFA in 0.1 M PBS (pH 7.4) for 3 h at RT; after washing in dH_2_O (3 × 5 min) specimen were post-fixed in 1% OsO_4_ in the same buffer for 1 h; rinsed again and dehydrated in ascending ethanol series, 30% 50%, 70%, 2 × 10 min, on a rotor, 96%, 100%, 2 × 15 min; then specimen were impregnated with resin LRWhite I, 4 h and RWhite II, 3 h, on a rotor at RT and polymerized in fresh LRWhite in gelatin capsules at 58°C for 24 h. The fixation protocol of tissue of adult* locusts (Schistocerca gregaria)* for immunogold-staining was described in Skiebe et al. (2006).

Semithin-sections for light microscopy (1.5 μm) and ultrathin sections (50–70 nm) for electron microscopy were cut on a Leica EM KMR2 ultramicrotome. For ultrastructural immunocytochemistry, the sections were mounted on gold-plated slots. The protocol of etching was the same as described in Skiebe et al. (2006) with using 2% periodic acid, saturated Na-metaperiodate and washing in dH_2_O. After etching, sections were washed in 1% sodium metabisulfite (SMBS) in dH_2_O. 2 × 10 min, and then in 0.1 M Tris-HCl, pH 7.0, + 0.1% SMBS + 0.05% NaN_3_, 10 min, RT.

Pre-incubation was done in 5% NGS in 0.1 M Tris-HCl (pH 7.0) + 0.1% SMBS + 0.05% NaN_3_ for 45 min at RT. Subsequently, sections were incubated in the primary monoclonal anti-octopamine antibody developed in mouse (Jena Bioscience), 1:500 in 0.1 M Tris-HCl, pH 7.0, + 0.1% SMBS + 0.05% NaN_3_ + 1% NGS for 18 h at 4°C on a shaker. After this sections were washed in 0.1 M Tris-HCl (pH 7.0) + 0.1% SMBS + 0.05% NaN_3_ for 3 × 10 min, then in 0.1 M PBS (pH 7.4) for 3 × 2 min, and then in 0.1 M PBS + 0.2% bovine serum albumin (BSA) for 3 × 2 min. Then, the secondary antibody was applied (goat anti-mouse-IgG-gold-conjugate, particle size 15 nm, AURION, Netherlands), 1:20 in 0.1 M PBS (pH 7.4) + 0.2% BSA for 2 h at RT on a shaker. Finally, sections were washed in 0.1 M PBS 5 × 5 min, then in dH_2_O 3 × 4 min before contrasted in 4% uranyl acetate (in dH_2_O) for 10 min at 37°C and in 0.4% lead citrate for 5 min at RT and examined with a Philips 208 transmission electron microscope.

### Behavioral Treatments

#### Starvation Experiments of Fruit Fly (*Drosophila*) Larvae

Wandering, still feeding early third-instar larvae were put on wet filter paper without food for up to 6 h and dissected after 2 h (*n* = 8), 4 h (*n* = 8) and 6 h (*n* = 8). Fed larvae served as the control group (*n* = 32). Each of the flies that were used in this study carried a copy of Tdc2-GAL4 and UAS-CD4-GFP. Larvae were dissected and immuno-stained as described earlier.

#### Synaptopod Quantification in Fruit Fly Neuromodulatory Axons

*Synaptopod* numbers of octopaminergic branches were measured manually at two body wall muscle fibers (14, 28) using the open-source software Fiji, a distribution of ImageJ version 1.48b (Schindelin et al., 2012). Branches, other than the “average base (main) branches”, were counted as *synaptopods* if they measured at least 5 μm in length (see Supplementary Figure S4).

### Blocking Release From Fruit Fly Type II Terminals by RNAi-Bruchpilot

#### Fly Care

Flies were raised at 25°C and 60% relative humidity with a 12 h/12 h light/dark cycle. The Tdc2-GAL4 (II) stock was kindly provided by Henrike Scholz, Cologne, and outcrossed into CantonS background. The w-UAS-brp-RNAi^B12^ (X) stock was kindly provided by Stephan Sigrist, Berlin.

#### Behavioral Experiments

After briefly immobilizing 2–3 days old female flies by cold-anesthesia, head and thorax were glued (Sinfony Indirect Lab Composite, 3M ESPE, St. Paul, MN, USA) to a triangle-shaped copper hook (0.05 mm diameter). The animals were then kept individually in small moist chambers containing a few grains of sucrose until testing 1 or 5 h later. The hook was clamped magnetically to a stand to accomplish stationary flight. The fly was surrounded by a homogenous panorama under room light conditions. The observer sat behind the setup and removed a polystyrene bead or a piece of filter paper from the fly’s tarsi. This initiated spontaneous flight and the experiment was started. The time until the fly stopped flying was recorded for three consecutive flights whereby 600 s was a given maximum of flight duration. When the fly ceased flying it was gently stimulated from the front side using a fly aspirator. The duration of the longest out of the three flights (see Figure 8A) or the duration of the first flight (see Figure 8B) were listed as a data point for each fly.

**Figure 1:**
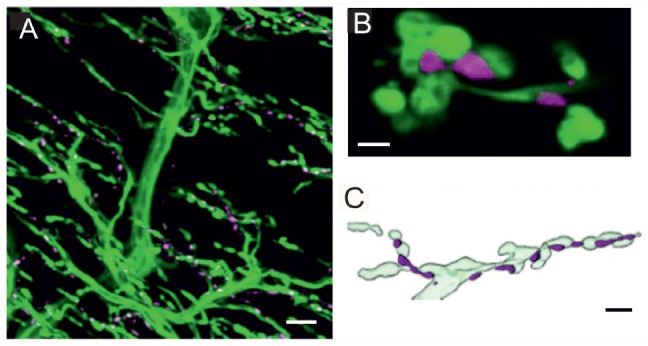
Innervation of locust (*Schistocerca gregaria*) pleuroaxillary muscle M85. **(A)** Neurobiotin-Streptavidine-Fluorochrome (green) labeled anterograde fill of the motor nerve (N4D4) of M85 and anti-octopaminergic labeled varicosities (magenta). Z-dimension of image stack 100 μm, scale bar 10 μm. **(B)** Individual octopaminergic boutons (magenta) lie very close to motor neuron terminals (green); the distance between both structures is less than 300 nm. Single optical slice of 1 μm width, scale bar 2 μm. **(C)** Schematic illustration of the typical arrangement of neuromodulatory octopaminergic varicosities (magenta) and the motor fiber terminals (light green). Scale bar 5 μm.

**Figure 2:**
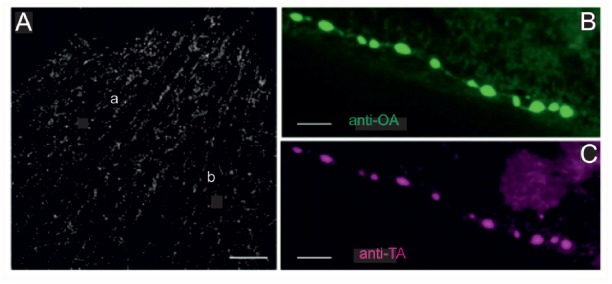
Locust (*Schistocerca gregaria*) pleuroaxillary (*“flight steering”*) muscle (M85) in which terminals are stained by octopamine- and tyramine- immunoreactivity. **(A)** OA-ir reveals evenly distributed chains of varicosities on muscle parts a and b of M85. Z-Dimension of image stack 30 μm, scale bar 100 μm. **(B,C)** Co-localization of OA-ir **(B)** and TA-ir **(C)** in boutons on M85 (same preparations). Z-Dimension of image stacks 11 μm, scale bar 5 μm.

**Figure 3:**
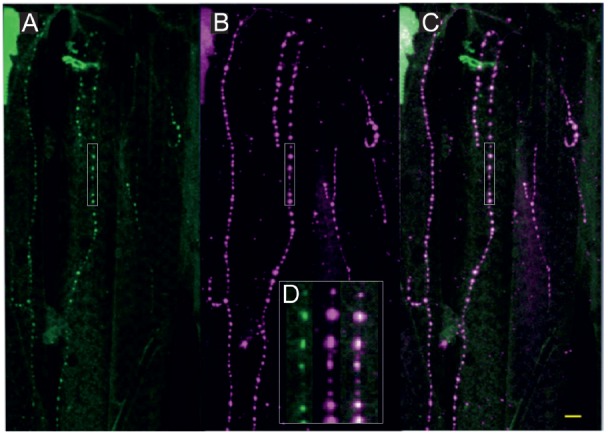
Fruit fly (*Drosophila melanogaster*) larva body wall muscles labeled with anti-tyramine **(A)** and anti-octopamine **(B)** reveals co-labeling in the type II terminals of the beaded fibers (composite **C**). Scale bar: 10 μm. **(D)** Type II terminals at higher magnification. The magnified area is depicted by a white rectangular box in **(A–C)**. Scale bar: 2.5 μm.

**Figure 4:**
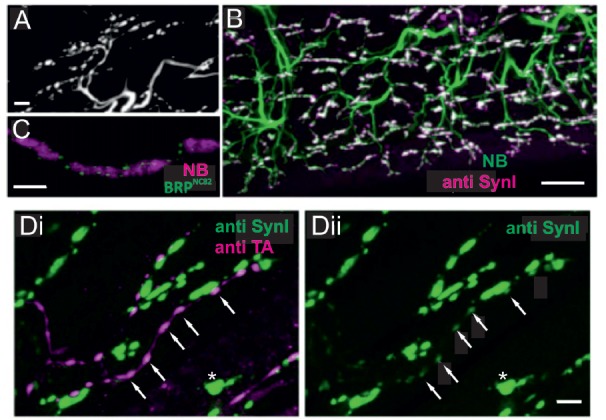
Locust (*Schistoceca gregaria*) motor neuron terminals on M85 stained with antibodies to synaptic proteins. **(A)** Typical appearance of motor neuron terminals on M85 labeled with neurobiotin by anterograde staining of N4D4. Terminals are aligned in parallel to fibers and cover the entire muscle. Z-Dimension of image stack 10 μm, scale bar 10 μm. **(B)** Labeling of M85 with anti-synapsin I (magenta) in addition to the backfilled motor nerve by neurobiotin (green) highlights the presence and distribution of presynaptic sites (white). Z-Dimension of image stack 30 μm, scale bar 20 μm. **(C)** Immunoreactivity to the synaptic protein bruchpilot (BRP), BRP^NC82^ (green), can be located in motor terminals (magenta, NB-labeled) as distinct puncta presumably representing active zones. Z-Dimension of image stack 30 μm, scale bar 5 μm. **(D)** Double-labeling with anti-synapsin I (green) and anti-tyramine (magenta) shows the characteristic beaded octopaminergic/tyraminergic fibers in proximity to motor terminals (green, **Di**). In addition to the markedly stained motor terminals by anti-synapsin (green, **Di** and **Dii**, one motor terminal marked by white asterisk) weak labeling in OA/TA-ir boutons is also revealed (white arrows in **Di** and **Dii**). Z-dimension of image stacks 10 μm, scale bar 5 μm.

**Figure 5:**
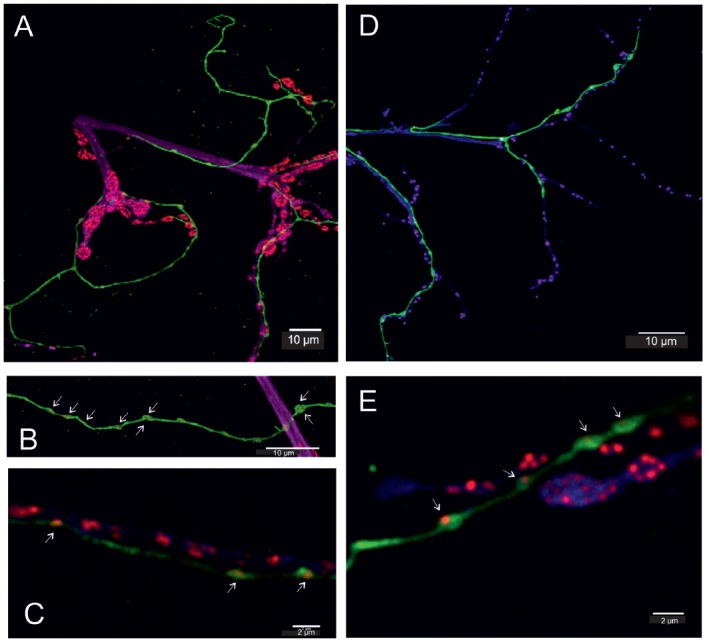
Fruit fly (*Drosophila melanogaster*) larva **(A–C)** and adult **(D,E)**. **(A–C)** In transgenic flies (TDC2-gal4 × UAS-CD8-GFP, green) all VUM-neuron axons are labeled in green. Additionally, motor axons of body wall muscles are labeled by anti-Horseradish peroxidase (HRP) in magenta/blue and active zones of synaptic sites of the neuromuscular junction (NMJ) are labeled by BRP^NC82^ (“anti-bruchpilot”) in red and are adjacent to the GFP-labeled (green) octopaminergic axons **(A)**. In the GFP-labeled octopaminergic fibers one or two BRP-labels are revealed in each bouton (varicosities in **B,C**, white arrows), and in **(C)** red BRP-labeling is also seen in the motor axon. **(D,E)** In the adult DLM flight muscle (FM) the axons of motor neurons are labeled by anti-HRP (blue) and again show their relationship to the VUM-neuron fiber stained in green and the red labels of the active zones. The structure of the NMJ of adult muscle differs from that of larval muscle, particularly good to see in **(E)**. However, the octopaminergic VUM-fiber like in larvae reveals one (or two) BRP-spots (punctae) in each bouton (varicosity). Scale bars:** (A,B,D)**: 10 μm, **(C,E)**: 2 μm.

**Figure 6:**
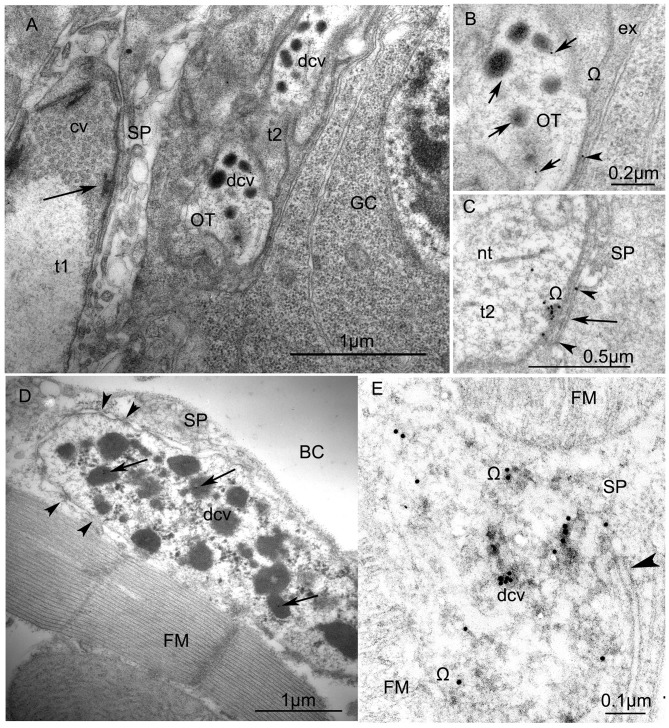
Ultrastructure of VUM-neuron terminals in larval **(A,B)** and adult fruit flies, *Drosophila melanogaster* **(C,D)**. **(A)** Arrangement of two terminals on the surface of a larval body-wall muscle: one synaptic terminal (t1) with clear vesicles (cv) has an “electron dense T-bar” (arrow) indicating the active zone, contacts with thin sarcoplasmatic processes (SP) and, thus, corresponds to the NMJ (or type I-terminal); the other type (t2) includes dense-core vesicles (dcv) with anti-octopamine Immuno-Gold labels, 10 nm, and corresponds to type II terminals. Other abbreviations: Glial cell (GC) with nucleus; OT, octopaminergic type II terminal (Scale = 1 μm). The immunogold labels are clearly depicted in **(B)** (arrows) which is a higher magnification of a part of the type II terminal shown in **(A)**. Abbreviation: ex, extracellular matrix; Ω, omega-profile on membrane of release-site. **(C)** shows a dense core vesicle in the process of exocytosis (arrow, see accumulated gold particles) and gold particles in the synaptic cleft (arrowheads). **(D)** An octopaminergic type II axon terminal with many dense-core-vesicles (dcv) on an adult *Drosophila* dorsal longitudinal FM labeled by 12 nm Immunogold-gold particles (arrows). The terminal makes desmosome-like contacts (arrowheads) with SP. Other abbreviation: body cavity (BC) (Scale =1 μm). **(E)** A higher magnification of anti-OT-immunogold labeling in the dense core vesicles and the release site (arrowhead) (Scale = 1 μm). This tissue was not contrasted by heavy metals to reveal 15 nm-Immuno-Gold labels.

**Figure 7:**
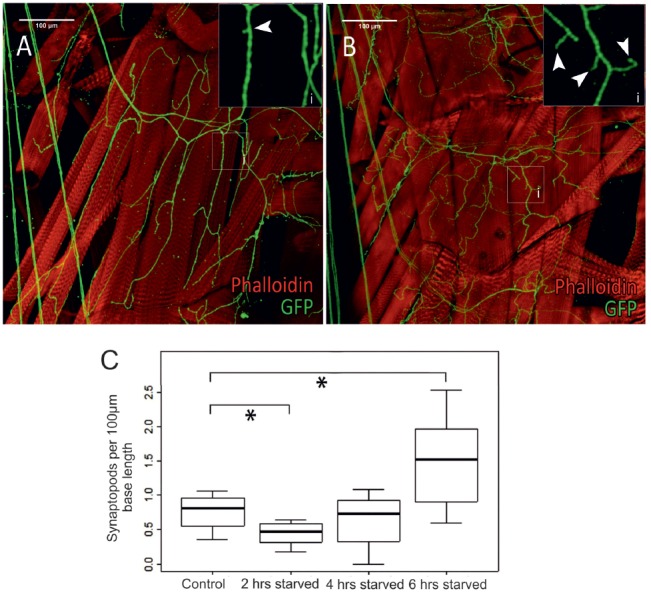
Differences in octopaminergic innervation by VUM-neuron axon (green) of *Drosophila* larval body muscle (14 and 28, below muscle fibers 6 and 7) between a control animal **(A)** and a larva that was starved for 6 h **(B)**. More octopaminergic branches are found in the starved animal compared to the control. **(Ai,Bi)** show magnified sections of areas marked by white squares in **(A,B)**, respectively. **(C)** Within 2 h of food deprivation the number of *synaptopods* decreases significantly. If starvation continues an increase in the number of *synaptopods* can be observed. After a total of 6 h of starvation the larvae have a significantly increased number of *synaptopods* (*n* = 32; *p < 0.05).

**Figure 8:**
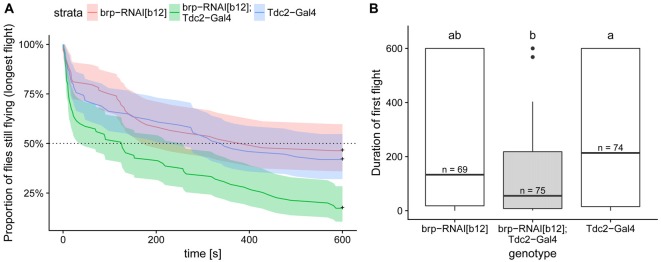
Expression of BRP-RNAi in octopaminergic and tyraminergic cells led to a decreased flight duration compared to the two genetic controls. **(A)** Kaplan Meier survival curve of the longest of the three first flights. This analysis, which is the most suited to this type of right-censored data, detect the difference between test and control flies (Cox proportional hazards regression model, corrected *p* < 0.004 with Bonferroni corrections). **(B)** Duration of the first flight is shown in order to compare the values with the one reported in Brembs et al. (2007). Boxplots represent the median (bar), the 75%- and 25%-quartiles (box) and all the residual data points (whiskers). Letters indicate statistically significant difference between groups (Wilcoxon rank sum test with bonferroni corrections for multiple comparisons, corrected *p* < 0.05). N-values are the number of animals examined. Data availability: behavioral data and its analysis are available at: https://doi.org/10.5281/zenodo.1160648.

#### Statistical Analysis

For survival analysis of flight duration, we used Kaplan-Meier curves and Cox proportional hazards regression model. For the duration of the first flight, we used a Wilcoxon rank sum test (the data don’t show normal distribution since values are truncated at 600 s). Bonferroni corrections were used for multiple comparisons.

## Results

### Octopaminergic Axonal Terminals in Locusts (*Schistocerca*)

In locusts, a well-studied wing muscle, the mesothoracic pleuroaxillary muscle M85 changes the angle of pronation of a forewing and, thus, is involved in flight steering. Muscle M85 consists of two parts and is innervated by two glutamatergic excitatory motor neurons (Pflüger et al., 1986), one GABAergic common inhibitor neuron (Wolf and Lang, 1994), and by an octopaminergic DUM3,4,5-neuron (Stevenson and Meuser, 1997). Figure 1A shows an anterograde neurobiotin fill of the motor nerve N4D4 which labels the axonal paths and terminals of all neurons innervating this muscle (green). The axonal terminals of the octopaminergic DUM3,4,5-neuron are also labeled with an α-octopamine AB (magenta) and appear very similar to the type II terminals of octopaminergic VUM-neurons of *Drosophila* larvae. The small neuromodulatory boutons are in close vicinity to the excitatory glutamatergic motor axons with large terminals corresponding to NMJs and in *Drosophila* terminology to type I terminals. The close spatial relationship between octopaminergic type II terminals and glutamatergic type I terminals is exemplified in a representative single optical section in Figure 1B, where the green labeled motor terminals encircle the magenta labeled neuromodulatory terminals of the DUM3,4,5-neuron. Figure 1C shows a sketch of the relation between motor and neuromodulatory axons in locust muscle and also schematically reveals the differences between motor axons forming NMJs or type I terminals and the neuromodulatory axons (*beaded fibers*) with their type II terminals.

Labeling of all octopaminergic axonal terminals on a whole locust muscle (M85) is shown in Figure 2A. Octopaminergic type II terminals seem to occur at a rather evenly distributed density and to form a dense meshwork through the entire muscle fibers with no obvious spared parts of the muscle. As octopamine is synthesized from tyramine and the dendritic profiles of DUM-neurons were shown to stain with both a tyramine- and octopamine-AB (Kononenko et al., 2009), we also tested whether OA and TA co-existed in the axonal type II terminals. As for the central profiles of DUM-neurons each axon terminal also expresses tyramine- and octopamine immunoreactivity (Figures 2B,C).

### Octopaminergic Axonal Terminals in Fruit Fly (*Drosophila*) Muscle

All neurons which synthesize tyramine/octopamine from tyrosine by using the enzyme tyrosine-decarboxylase (TDC2) can be visualized in fruit flies (*Drosophila melanogaster*) by targeted expression of UAS-mcd8GFP under the control of TDC2-gal4. Most larval muscles are supplied by these neuromodulatory neurons which, in *Drosophila*, belong to the class of ventral unpaired median (VUM-) neurons (Bräunig and Pflüger, 2001; Vömel and Wegener, 2008; Busch et al., 2009; Busch and Tanimoto, 2010; Koon et al., 2011; Selcho et al., 2014). In *Drosophila* larvae all VUM-neuron axons also appear as *beaded fibers* with varicosities or boutons in regular distances that are classified as type II terminals (Monastirioti et al., 1995; Sinakevitch and Strausfeld, 2005). Figure 3 shows that, like in locusts (see Figures 2B,C), these axon terminals express both tyramine- and octopamine-ir (see Figure 3D).

### Octopaminergic Axonal Terminals in Locusts (*Schistocerca*) Contain Major Presynaptic Proteins

Synaptic vesicle exo- and endocytosis and the underlying networks of interacting proteins are particularly well studied at excitatory NMJs in *Drosophila* larvae (Haucke et al., 2011; Südhof, 2012, 2013; Kononenko and Haucke, 2015) but less so in adult insects including fruit flies and neuromodulatory type II terminals. Therefore, we tested in locust adult muscle whether some of the key cytomatrix proteins known from type I axon terminals of motor neurons were also present in the axonal type II terminals of neuromodulatory neurons. In Figure 4 labeling with a synapsin-AB (Klagges et al., 1996) and with the active zone marker BRP^NC82^ (BRP; Wagh et al., 2006; Wichmann and Sigrist, 2010), reveals synaptic sites on locust muscle M85. An anterograde fill of the excitatory motor axons to M85 with neurobiotin reveals large axon terminals which run parallel to the muscle fiber length and exhibit the typical morphology of type I terminals (Figure 4A). In Figure 4B an overlap of such an anterogradely stained motor nerve (green) with an antibody staining against synapsin (magenta) reveals the many presynaptic sites (composite color white) of such an adult insect muscle. Labeling with the NC82-antibody (Figure 4C) also shows a “puncta”-distribution (green) on the large axon terminals (magenta), most likely indicating the active zones of presynaptic sites. In Figures 4Di,Dii simultaneous immunostainings with synapsin-AB (green) and a tyramine-AB (magenta) are shown. Figure 4Di proves that the *beaded fibers* with varicosities are the ones belonging to the neuromodulatory neuron which contains both tyramine and octopamine (white arrows, see also Figures 2B,C). In addition to the large motor axon (type I) terminals the varicosities of *beaded*, tyraminergic/octopaminergic *fibers* also express synapsin-ir (white arrows in Figure 4Dii). Please note that synapsin positive puncta are considerable smaller and less bright in type II terminals as compared to type I terminals (Figure 4Dii).

### Octopaminergic Axonal Terminals in Fruit Flies (*Drosophila*) Contain Major Presynaptic Proteins

In addition to type I motor axon terminals, also type II terminals of neuromodulatory VUM-neurons contain the active zone marker BRP (Figure 5). Figure 5A shows the GFP-labeled axon of a VUM-neuron (green) in the vicinity of NMJs of the motor axons stained in blue/magenta on a larval body-wall muscle. The presynaptic active zones are revealed by BRP^NC82^ (BRP, red) and show their typical circular (*“donut”-shaped*) arrangement. Magnifications of the VUM-neuron axon (green) shown in Figures 5B,C reveal small BRP^NC82^ active zones in type II terminals (see white arrows pointing to red punctae). Again, the close vicinity of the octopaminergic VUM-neuron axons with the much larger terminals of the type I motor axons is obvious, in particular in those preparations in which the motor axons were additionally stained by using an HRP-antibody. In contrast to the type I terminals with many active zones, the varicosities or boutons of the VUM-neuron axon possess only one or two active zone each (Figures 5B,C, see two varicosities with two red punctae in Figure 5B, white arrows). In adult muscle (Figure 5D), again the GFP-labeled VUM-neuron axon accompanies the motor axons marked in blue by anti-HRP. NMJs in adult muscles are different to their counterparts in larvae as they are much less pronounced and much smaller (compare Figure 5A with Figure 5D). Nevertheless, they also show labels of BRP^NC82^ (red) indicating BRP distribution in presynaptic active zones. A magnification in Figure 5E clearly shows that also in adult type II terminals one spot (puncta, white arrows) exhibits BRP^NC82^, in contrast to the larger and more numerous BRP^NC82^ labeling (red) of the motor terminals (blue). In the Supplementary Figure S1 BRP^NC82^ (BRP) labeling is also revealed in the central, dendritic parts of octopaminergic VUM-neurons indicating that VUM-neurons may also possess presynaptic release sites within the ganglia of the ventral nerve cord.

### Ultrastructure of Octopaminergic Axon Terminals in Locusts and Fruit Flies Including Immunogold-Stainings

The micrographs in Figure 6 show the ultrastructure of axon terminals of octopaminergic VUM-neurons in larval (Figures 6A,B) and adult (Figures 6C–E) fruit flies (*Drosophila melanogaster*). In particular, the micrograph in Figure 6A shows the differences in structure of a type I (NMJ, t1) and a type II (octopaminergic, OT, t2) axon terminal. Whereas the type I terminal is characterized by many clear vesicles and the presence of a clear active zone depicted by the electron dense T-bar (black arrow in Figure 6A), the type II terminals only contain dense core vesicles (dcv) and no T-bar (Figures 6A–C). This was found in all animals tested. Identification of OA positive profiles was accomplished by immuno-gold coupling to an α-octopamine antibody (see black arrows in Figures 6B,C). Similar type II terminals full of dense core vesicles are present on the adult flight muscle (FM, Figure 6D). The inserts in Figures 6B,C,E show anti-octopamine immunogold labeling in dense core vesicles, or in the process of exocytosis from a dense core vesicle (arrow in Figure 6C). These ultrastructural studies combined with immunogold-methods unequivocally show that octopamine is stored and released from dense core vesicles (Figures 6C,E). In the Supplementary Figure S2 more immunogold-labeling is shown and there are indications based upon the distribution of gold-particles that once octopamine is released into the extracellular space it may be taken up by glial cells (GCs) from where it may be recycled (Ryglewski et al., 2017).

### Plasticity of Tyraminergic/Octopaminergic Axon Terminals in the Fruit Fly Larvae After Starvation

In a series of elegant live imaging experiments of axon terminals of VUM-neurons in *Drosophila* larvae, Koon et al. (2011) found that starvation for 2 h induced an activity dependent growth of octopaminergic *synaptopods* or small axonal sprouts with newly forming output synapses (see also Supplementary Figure S4), and that this is controlled by a cAMP- and CREB-dependent positive-feedback mechanism requiring Octb2R autoreceptors. This autoregulation was necessary for the observed increased locomotor responses after starvation. In order to further investigate the temporal dynamics of this process we examined type II terminals of selected body wall muscles (Figure 7A) in transgenic TDC2gal4 × UASCD8GFP larvae starved for 2, 4 and 6 h (Figure 6B) and measured the *synaptopods* per 100 μm (Figure 7C). In addition to the previously reported starvation induced growth of aminergic *synaptopods* after 2 h of starvation (Koon et al., 2011), we observe a temporal dynamic with an initial decrease in the number of *synaptopods* followed by an increase after 6 h of starvation.

Activity-dependent plasticity of octopaminergic type II terminals can also be observed in locust muscle. In the supplementary material more information can be found including Supplementary Figure S3.

### The Effect of Blocking Release From VUM-Neuron Terminals on Flight Performance of Adult Fruit Flies (*Drosophila melanogaster*)

This and previous studies demonstrate the existence of similar molecular components of the synaptic vesicle release machinery in type II and type I terminals. However, for type II terminals it has not yet been tested whether molecules, such as the CASK protein BRP, are critically required for synaptic vesicle release as is the case in type I terminals (Wagh et al., 2006). It had been previously shown that flies deficient in octopamine synthesis (Monastirioti et al., 1995) or deficient of octopaminergic/tyraminergic neurons exhibit deficits in flight performance (Brembs et al., 2007). In order to test whether we could block OA release by disturbing the BRP-dependent vesicle release mechanisms, we expressed a potent UAS-RNAi transgene (brp-RNAi^B12^) under the control of the tdc2-Gal4 driver. As expected, control flies are flying well in our assay. In contrast, the test flies stopped flying much earlier than their controls (Figure 8A). This phenotype was reminiscent of the effect of eliminating these neurons reported in Brembs et al. (2007) compare Figure 8B here to their Figure 5A). Unfortunately, differences in the performance of the control flies between the two studies, probably due to differences in fly care protocols, make a direct comparison of the two sets of data difficult. But our results strongly suggest that down-regulating of BRP in octopaminergic/tyraminergic neurons impairs OA release from type II terminals.

## Discussion

The evolutionarily old hemimetabolous orthopteran desert locust, *Schistocerca gregaria*, and the evolutionarily more recent holometabolous dipteran insect, *Drosophila melanogaster*, possess octopaminergic/tyraminergic neurons with highly conserved features. In both species OA/TA neurons have unpaired cell bodies along the dorsal (DUM neurons, locust) or ventral (VUM-neurons, fruit fly) midline and bilaterally symmetrical axons that project through efferent nerves (Bräunig and Pflüger, 2001; Pflüger and Stevenson, 2005). In addition to morphological similarities these neurons share multiple physiological and functional features (see Bräunig and Pflüger, 2001).

In the thoracic ganglia these VUM- or DUM-neurons are efferent cells that send their axons to a variety of target tissues including skeletal and visceral muscles, glands and sense organs. Clearly, in both insects the axon terminals of octopaminergic neurons share many commonalities: (i) they form boutons or varicosities in regular distances across the axon and, thus, give them a “beaded appearance” (*beaded fibers*). These characteristic axon terminals have been named type II terminals in *Drosophila*. In contrast to the type I terminals that represent the NMJ formed by excitatory motor neurons, the synaptic contacts made by the octopaminergic neurons are those of “en-passant-synapses” lacking clear pre- and postsynaptic specializations at an ultrastructural level. The idea from this study is that neuromodulators are released into a “volume space” whose borders may be depicted by GCs, trachea, extracellular matrix or other, more specialized barriers, similar to what is discussed for the efficacy of NO (Münch et al., 2010).

### Type I- and Type II-Terminals Share Common Release Mechanisms

In locusts, the vesicular calcium sensor synaptotagmin is present in both the excitatory NMJ (type I-terminal) and the neuromodulatory (octopaminergic) *beaded fibers* (type II terminals), a finding which is also supported by studies of the octopaminergic unpaired median neurons in the tobacco hornworm (*Manduca sexta*; Consoulas et al., 1999). In larval and adult *Drosophila* muscles, the active zone protein BRP is also present in both type I and type II terminals although the density of active zones is very different. Whereas the type I terminals or NMJs reveal many active zones, the type II terminals only reveal one or two, maximally three sites of active zones that may easily be overlooked in preparations. Similarly, at the ultrastructural level a classical T-bar is found in the active zone of type I terminals but not in type II terminals. However, the presence of two of the many synaptic proteins in both types of terminals may indicate similar molecular mechanisms for synaptic vesicle release at type I and type II terminals. But note the fact that the NMJ of excitatory glutamatergic motor neurons only contains clear vesicles, whereas we found only dense core vesicles in type II terminals. This is in line with the previous, most elegant study of Koon et al. (2011) in which they also show similarities in release mechanisms of type I and type II terminals. In addition to BRP and synaptotagmin, the synaptic reserve pool protein synapsin is also present in both type I and type II terminals, for example also nicely illustrated in the locust antennal heart (Antemann et al., 2018). This may indicate the existence of reserve and release pools in axonal terminals which only contain dense core vesicles, similar to what is described for terminals with clear vesicles.

Interesting observations can be made by combining ultrastructural studies with immunogold-staining (anti-octopamine). Octopamine is indicated by the distribution of gold-particles which were found clustered in dense core vesicles, less dense in extracellular space close to what is regarded the “synaptic cleft” and a release site (Ω profile) of a type II terminal and also scattered within GCs (see also Supplementary Figure S2). A classification of terminals other than into type I and type II was used by Jia et al. (1993) who distinguished clear vesicle boutons, dense core vesicle boutons and “mixed” vesicle boutons. They report that dense core vesicle boutons also contain small translucent vesicles (33 ± 0.5 nm) which we did not find in our study of octopaminergic type II terminals. In agreement to our results, they also report that release sites in the dense cores vesicle boutons look very different to those of the clear vesicle boutons (which correspond to the NMJ).

### Neuromodulatory Type II—Terminals Are Very Dynamic

The morphology of type II axon terminals are not static, but by contrast can be adaptively altered in response to changing conditions. It has been shown that starvation for 2 h causes the formation of additional small branches which were called “*synaptopods*” (Koon et al., 2011, and Figure 7). Our data further confirmed that type II terminals undergo plastic morphological changes. However, we gathered slightly different results with regard to the timing and the net effect of starvation induced changes of type II terminal morphology. In our study, an initial decrease in the number of *synaptopods* after 2 h of starvation was followed by a marked increase after 6 h of starvation. This discrepancy could be caused by slightly different ages of the animals at the onset of starvation (for example early vs. late third-instar wandering larvae), differences in diet, or different modulatory backgrounds. Our data indicate a more dynamic regulation process, probably involving positive- as well as negative-feedback mechanisms (Mathejczyk, 2013). The decrease in *synaptopod* numbers within the first 2 h of starvation may be due to activation of Octß1R autoreceptors during that time (Koon and Budnik, 2012). Such a negative-feedback system may serve as a natural buffer for avoiding spontaneous stress responses and *synaptopod* formation every time the larva is without food for a short amount of time.

Dynamic changes after stressing stimuli can also be observed in locust octopaminergic terminals as depicted in the supplementary information including Supplementary Figure S3.

### Octopamine Release From Type II Terminals Is Necessary for a Normal Performance of Flight Behavior in Adult Fruit Flies

In a previous study (Brembs et al., 2007), fruit flies mutant for octopamine showed significant deficits in their flight performance. As we could show that type II terminals also contain the active zone protein BRP, we tested fruit flies in which the release from octopaminergic type II terminals was blocked by an RNAi-construct. The two main hypotheses explaining the presence of the canonical vesicle release mechanisms proteins at the VUM-neuron synapse are that either OA is released via this mechanism or that other neurotransmitters are present in these neurons. In order to test these hypotheses, we chose an OA-dependent Behavioral trait, flight performance. We tested the effect of a perturbation of BRP in OA neurons on that behavior. We know that RNAi perturbation blocks the common synaptic release (Wagh et al., 2006) and that ablating OA neurons perturbs fly flight. Our data show that RNAi induction in tyraminergic/octopaminergic neurons affects flight performance to a similar level like killing the neurons or preventing the production of OA. We therefore conclude that OA is indeed released via the common, BRP dependent, vesicle release mechanism. However, at present we cannot distinguish between release mechanisms centrally from dendrites or peripherally from axonal type II terminals of OA neurons as also the dendritic release sites of VUM-neurons contain BRP (see Supplementary Figure S1) and also label with both the tyramine- and octopamine-antibodies (Kononenko et al., 2009). In addition, ultrastructural studies of octopaminergic DUM-neurons in locusts show that the dendrites also seem to have release sites and that presynaptic electron-dense structures which clearly differ from the T-bar of NMJs are present (Watson, 1984; Pflüger and Watson, 1995). Thus, it cannot be excluded that DUM- or VUM-neurons also release octopamine and/or tyramine in the central neuropile.

## Conclusion

The results of this study show that the axons of the octopaminergic VUM-neurons in evolutionarily far apart insects such as locusts and fruit flies form *beaded fibers* with type II terminals which are closely associated with the motor axons forming “classical” NMJs or type I terminals. The type II terminals with their dense core vesicles also possess BRP as an important presynaptic cytomatrix protein of the Active Zone although the spatial arrangement must be different. The structure of the cytomatrix and the protein-protein interactions in type II terminals necessary for transmitter release from dense core vesicles has yet to be revealed. In addition, as far as we know all VUM-neurons in *Drosophila* persist through metamorphosis, and subsequently innervate adult muscle. There are indications that interactions between motor and neuromodulatory neurons occur during development (Vonhoff and Keshishian, 2017) and that these interactions are also important for forms of peripheral plasticity, for example after starvation (Koon et al., 2011) including octopamine signaling via different octopamine receptors (Koon and Budnik, 2012). However, the precise interactions between motor (type I) and neuromodulatory (type II) terminals during normal development and during metamorphosis in the pupal stages are less well studied. Last but not least many neuromodulatory neurons, for example those releasing serotonin and dopamine, possess similar type II terminals and, therefore, the study of octopaminergic type II terminals in *Drosophila* may yield important, more general insights into their release mechanisms.

## Ethics Statement

All experiments comply to the respective German laws on experiments on insects.

## Author Contributions

JC, CDuch, SS and H-JP designed the experiments and BS, CB, CDamrau, TM, CW and NR carried out parts of the experiments. NMB carried out all ultrastructural work and wrote the respective sections. HW was involved in experimental work and provided expert technical assistance. Some of the results presented here were parts of a PhD-thesis by BS and CDamrau, a bachelor and master thesis by TM, and a bachelor thesis by CW at Freie Universität. All authors contributed to parts of the manuscript which was written by CDuch and H-JP.

## Conflict of Interest Statement

The authors declare that the research was conducted in the absence of any commercial or financial relationships that could be construed as a potential conflict of interest.
