# Supplementary material for: Structural and Molecular Properties of Insect Type II Motor Axon Terminals
Source: Front Syst Neurosci. 2018 Mar 19;12:5. doi: 10.3389/fnsys.2018.00005 (PMC5867341; doi:10.3389/fnsys.2018.00005)
Supplement: Supplementary file 1 [file Presentation_1.pdf]

Suppl. Figure S1

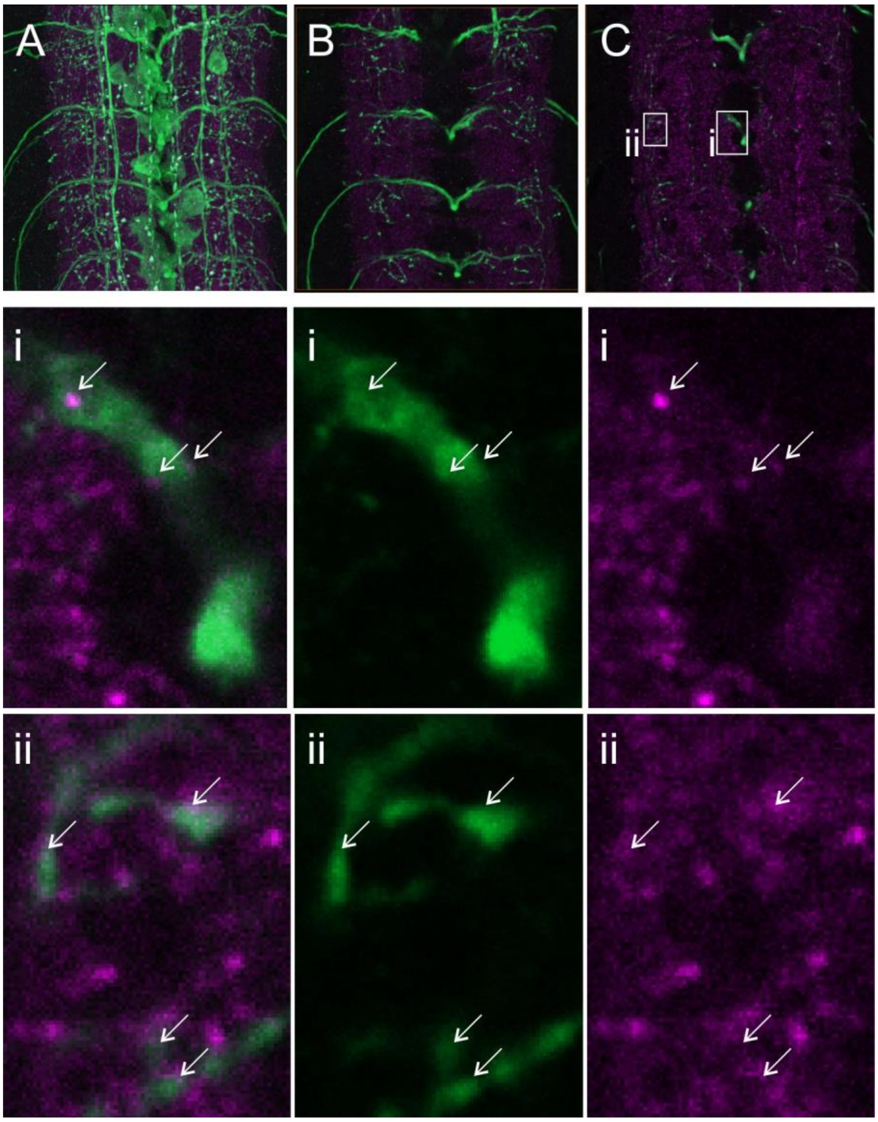

Suppl. Figure S1

(A) Projection view of confocal image stack of a representative double label for TDC2 positive VUM-neurons (green) and anti-brp immuno-label (magenta) in the larval *Drosophila* ventral nerve cord. (B) Projection view of 10 optical sections that show bifurcating VUM-neuron neurites and some smaller central processes of TDC2 expressing neurons in a 10 µm thick layer of the larval ventral nerve cord. (C) Single optical section of the same preparation as in A and B. The white boxes i and ii are selectively enlarged in the rows i and ii respectively. Micrographs (i) show three brp-positive puncta (white arrows) in the VUM-neuron neurite just distal to the bifurcation. This may indicate active zones in VUM-neuron central processes, or alternatively, transport of brp-positive puncta to the axon terminals in the periphery. (ii) shows-brp positive puncta (white arrows) in small dendritic processes of VUM-

neurons in the larval ventral nerve cord. This may indicate active zones and thus release sites in central processes of TDC2 positive neurons in Drosophila larvae.

Suppl. Figure S2

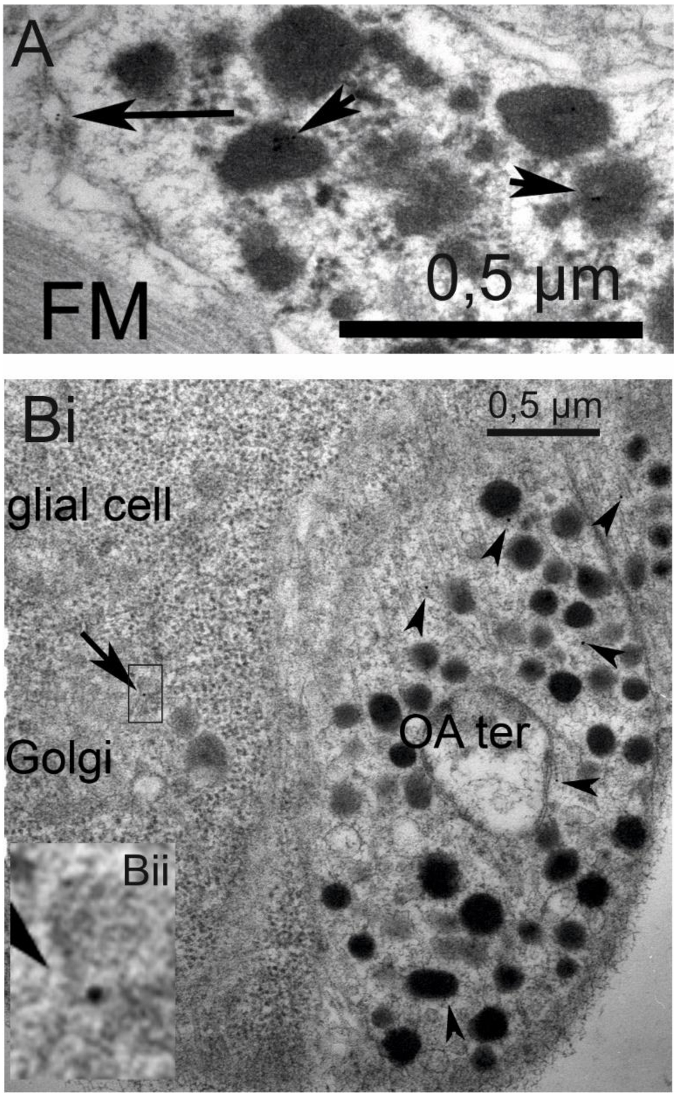

Suppl. Figure S2

(A) Ultrastructure of type II terminal on adult Drosophila flight muscle (FM). Small arrows point to immunogold labeling (10 nm) of anti-octopamine in dense core vesicles. Long arrow refers to gold particles in the extracellular spaces between two membranes (sarcolemma and neurilemma) as a result of release from a dense core vesicle indicated by the Ω-profile. (B) 10 nm immunogold-labels of anti-octopamine (arrowheads) in octopaminergic type II terminal (OA ter) on Drosophila larval body wall muscle. The arrow in Bi refers to one immunogold-label in the adjacent glial cell (see black rectangular) shown at much higher magnification in

Bii. This may indicate that glial cells may be involved in octopamine recycling once it had been released from type II terminals.

Suppl. Figure S3

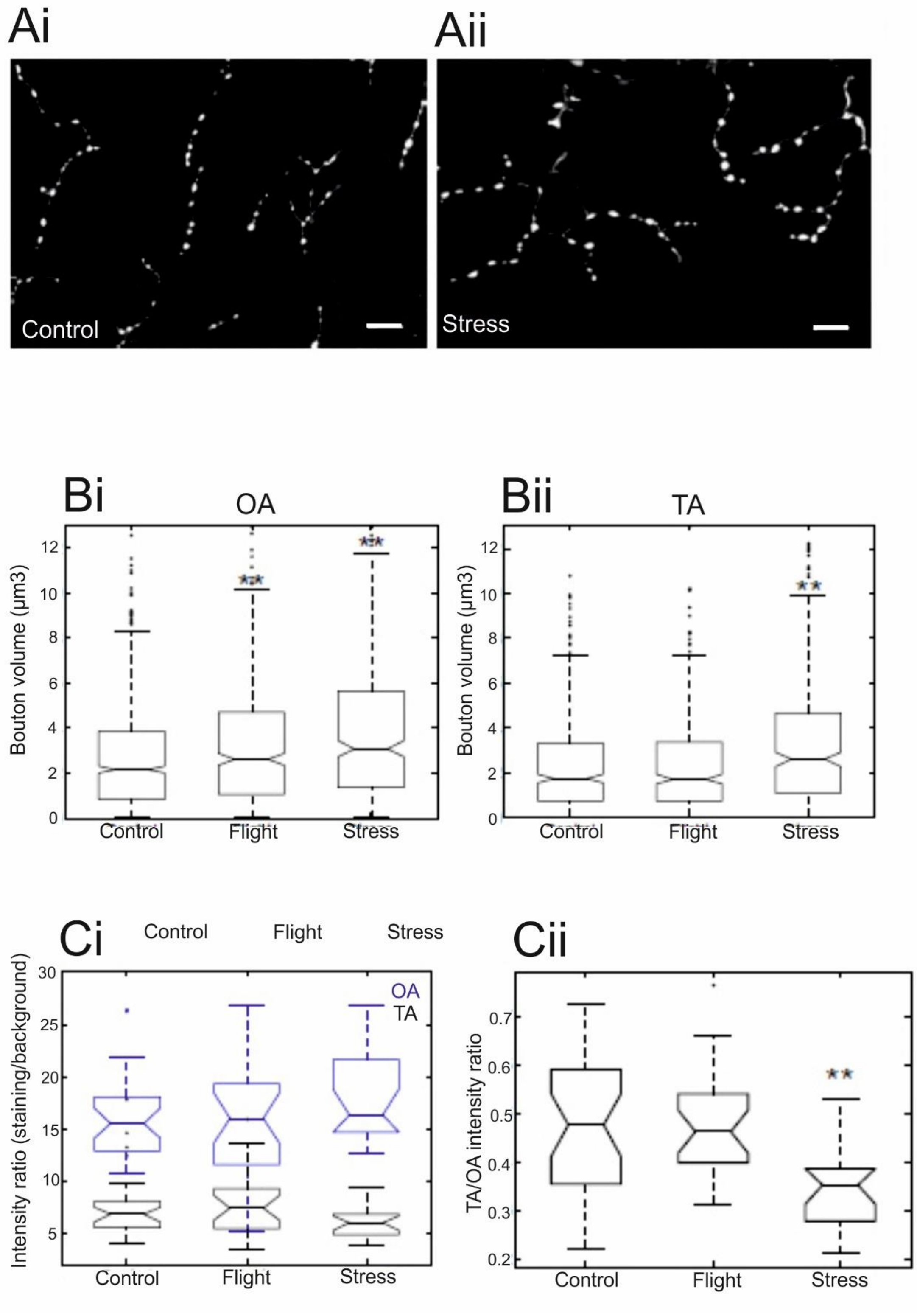

Suppl. Figure S3

Size of OA- and TA-immunoreactive boutons on M85 in dependence of behavioral experience. (A): Representative confocal image stacks display bouton appearance of control (Ai) and stress-exposed animals (Aii). Z-dimension of image stacks 11 µm, scale bar 10 µm. (B): Boxplots confirming significant differences between the octopaminergic (Bi) and tyraminergic (Bii) bouton volume distributions. Kruskal-Wallis-Test, **$p<0.01$. Number of boutons examined (OA/TA): Control=423/262, Flight=225/212, Stress=183/158. (C): The relative OA/TA-ir intensities of control and behaviorally treated groups reveal no differences among groups, although the signal-to-noise ratio of OA-ir in boutons (blue) is generally higher than that of TA-ir in boutons (black) (Ci). However, the ratio of these relative intensities (TA/OA) is significantly smaller for stress-exposed animals than for the control and flight groups (Cii). Number of image stacks examined: Control=33, Flight=26, Stress=19. Kruskal-Wallis-Test, **$p<0.01$.

Detailed description of results to Fig. S3: Plasticity of tyraminergic/octopaminergic axon terminals in the adult locust (Schistocerca gregaria) as caused by behavioral context.

To investigate whether the peripheral neuromodulator amounts measured by OA- and TA-ir were affected by the behavioral state of the animals, adult locusts of either sex were separated into three groups and subjected to different treatments. The control group (N=6) was left undisturbed, whereas the other two were either tethered to perform steady flight for 30 min to up 1 h (N=5) or exposed to stress for 15 to 20 min (N=4, see Methods) prior to tissue fixation and subsequent immunocytochemistry. Fig. S3 A shows representative anti-OA labels of type II terminals on muscle tissue of control (Fig. S3 Ai) and stress treated (Fig. S3 Aii) animals. A quantitative analysis of octopamine- and tyramine-labels in boutons was performed by selecting representative chains of stained varicosities from each muscle examined (Number of muscles examined: Control: N=9, Flight: N=8, Stress: N=7) and measuring their volume threshold-dependently with respect to the grey value histogram for distinguishing background from staining (see Methods below). Figs. S3 B,C show (boxplots of) the distributions of octopaminergic (Fig. S3 Bi) and tyraminergic bouton volumes (Fig. S3 Bii). The median value of octopaminergic bouton volumes within the control group was 2.2 μm^3. Significantly different medians were measured with respect to the stress-exposed group which exhibited the largest differences (median 2.57 μm^3) to the control group (Fig. S3 Bi). This corresponded well to the average bouton diameter of 1.7 µm in consideration of the volume of an ellipsoid body $V_e = 4/3\pi r_1 r_2 r_3$, where πr_1-r_3 designate the respective half

diameters. Significantly different medians were measured in with respect to the stress-exposed group which exhibited the largest differences (median 2.57 μm^3) to the control group (Fig. S3 Bi). The same held true for tyramine-ir boutons, where the boutons of stress-exposed animals again showed a significant shift towards larger boutons (median stress 2.4 μm^3 versus control 1.9 μm^3, Fig. S3 Bii). With octopamine-ir, significant changes in bouton size were also observed for flight (Fig. S3 Bi) but not for the sizes of tyramine-ir boutons (fig. S3 Bii). This is also depicted in Fig S3 C, where intensity ratios between immunoreactive staining and background (Fig. S3 Ci) or between octopamine-ir and tyramine-ir were measured (Fig. S3 Cii). The largest differences always occur within the stress group.

Methods to Fig. S3

Steady flight performance and stress exposure in locusts.

Animals of both sexes were either kept calm (n = 6, control group), exposed to different disturbing stimuli (such as noise and visual stimuli) for 15 to 20 minutes inside a plastic box (n = 4, stressed group), or kept flying for 30 min up to 1 hour (n = 5, flight group). Steady flight was enabled by gluing (Pattex) a light metal harness to the locust pronotum and linking it to a stand via a thin flexible plastic tube. Lack of tarsal contact to the ground and frontal exposure to a warm and perturbed air stream elicited flight in otherwise untethered animals. After the respective time interval the muscles were quickly dissected and fixed immediately as described in the chapter "*immunocytochemistry of locust muscle*". Exposure to stressing stimuli was achieved as described in Kononenko et al. (2009).

Labeling and evaluation of OA- and TA-immunofluorescent boutons in locusts

Image stacks obtained from confocal microscopy with "beaded" fibers on locust muscular tissue (M85) were preprocessed to correct for slight intensity gradients caused by the image acquisition process (module "correct z-drop" in Amira 4.1.1) and to reduce background staining (median filter, rolling ball algorithm in ImageJ, open source software). One chain of varicosities per image stack was selected and labeled semi-automatically using the "labelfield" mode in Amira. The threshold for accepting voxels (0,15 x 0,15 x 1 µm) as immune-positive staining corresponded with the steepest slope within the grey value histogram, indicating the transition from background to specific immune-positive staining. This value was correlated with an automatically assessed threshold value reflecting the 10 % brightest voxels per stack, which represented a means to correct for slight intensity differences between image stacks (samples were processed in a random mode to exclude

personal bias). Bouton volume data were then obtained from the Amira table "Tissue Statistics". Additionally, from each labeled image stack the mean grey value data of the so-determined background and specific staining were extracted and used for the comparison of intensity ratios. Diagrams were designed and statistics (nonparametric tests) performed with MATLAB R2007b (The Mathworks).

Suppl. Figure S4

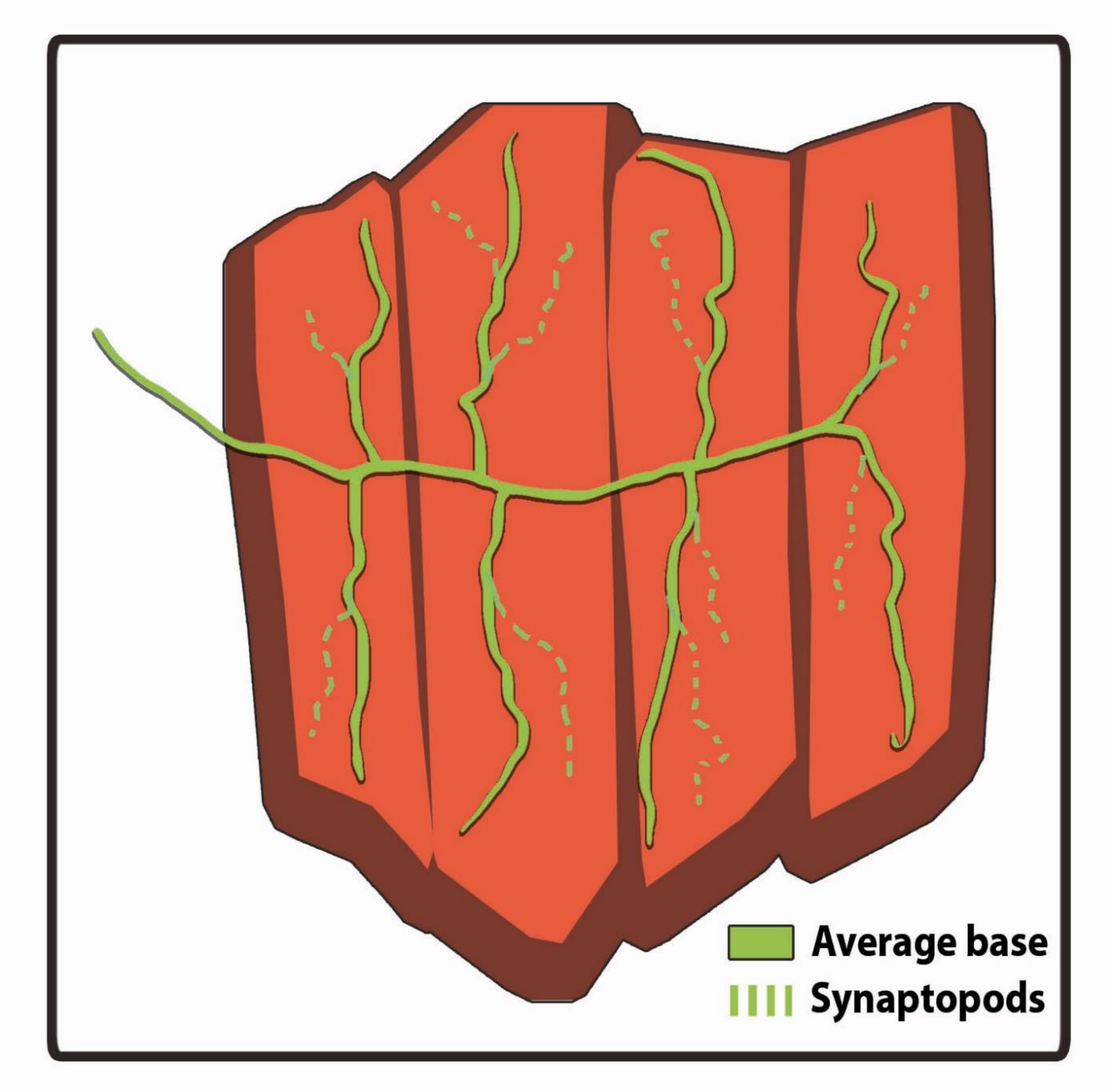

Suppl. Figure S4

This figure shows a schematic drawing of four larval body wall muscles innervated by the GFP-labelled octopaminergic axon of a VUM-neuron. The main, or “average base” axon is depicted as a solid green line, whereas the *synaptopods*, or axonal sprouts, are drawn as green dashed lines.
